# At Last, a Sensitive Method to Detect Incipient Systolic Dysfunction!

**DOI:** 10.5935/abc.20190198

**Published:** 2019-10

**Authors:** Carlos Eduardo Suaide Silva

**Affiliations:** 1 Diagnósticos da América SA, São Paulo, SP - Brazil

**Keywords:** Heart Failure, Ventricular Dysfunction Left, Cardiomyopathy, Hypertrophic, HIV, Antiretroviral Therapy Highly Active, Myocardial Contraction, Echocardiography/methods

Left ventricular systolic function evaluation has always been one of the main attributions of echocardiography. The degree of ventricular systolic dysfunction is an important predictor of outcome for a large number of diseases, including ischemic heart disease, cardiomyopathy, valvular heart disease, and congenital heart disease. In this area, the ejection fraction has been sovereign for many decades, but despite being a parameter that can, in most cases, inform the real state of global ventricular function, in many situations it may be normal in the presence of evident systolic or diastolic dysfunction.

Known clinical examples are the several cases of heart failure with preserved ejection fraction (HFpEF) or patients with hypertrophic cardiomyopathy, among others. For this reason, an index that can identify early ventricular dysfunction that is not connected with the ejection fraction has been sought for a long time.

Myocardial deformation evaluation by echocardiography emerged with the strain and strain rate techniques, still derived from tissue Doppler, which was developed at the Norwegian University of Science and Technology in Trondheim, Norway, approximately twenty years ago.^1,2^ In 2004, we had already demonstrated the presence of incipient systolic dysfunction in patients with the indeterminate form of Chagas disease using this technique.^3^ Later, in 2006, the speckle tracking technique appeared, which quantifies myocardial deformation by two-dimensional echo and does not depend on the insonation angle (a limitation of the prior technique).^4^This technique has advanced to the present day and allows measuring the longitudinal, radial and circumferential deformation of the several myocardial segments (strain). The mean value of the percentage of longitudinal deformation of each segment is what we call the global longitudinal strain (GLS) and this index has shown to be an excellent parameter for systolic function evaluation, which is sensitive enough to detect incipient impairment when the ejection fraction is still normal and has a higher prognostic value than EF in many clinical situations.^5-7^

In this issue of the Brazilian Archives of Cardiology, Dr. Ronaldo Campos Rodrigues presents us with an excellent work, in which left ventricular systolic function was assessed by quantifying GLS in HIV-positive patients, with and without antiretroviral therapy, compared with a control group.^8^ It was observed that GLS values were significantly lower in infected individuals than in control ones, regardless of whether or not they were undergoing treatment. All of them had normal ejection fraction and the only group with abnormal GLS (less than -18%) was that of infected individuals without treatment. Their findings demonstrate the high sensitivity of this echocardiographic parameter in detecting incipient systolic dysfunction, which leads us to think that treatment must decrease myocardial aggression by the virus. Early systolic dysfunction was also found by Mendes et al.,^9^ in a similar group.

These and other studies point to a paradigm shift in the study of ventricular function. I believe that soon, cardiologists will not be satisfied with the value of the ejection fraction alone but will also require the value of GLS for a deeper and more accurate assessment of ventricular systolic function.
